# Association Between Glucagon-Like Peptide-1 Receptor Agonists and Major Adverse Cardiovascular Outcomes Based on Race and Sex Among Patients With and Without Diabetes Mellitus: A Meta-Analysis of Nine Randomized Controlled Trials

**DOI:** 10.31083/RCM45797

**Published:** 2026-01-21

**Authors:** Vikash Jaiswal, Yusra Mashkoor, Vamsikalyan Borra, Asmita Gera, Nirmit Patel, Sahas Reddy Jitta, Yusra Minahil Nasir, Prachi Sharma, Jishanth Mattumpuram

**Affiliations:** ^1^Department of Cardiology, Endeavor Health Cardiovascular Institute, Glenview, IL 60201, USA; ^2^Department of Internal Medicine, Dow University of Health Sciences, 74200 Karachi, Pakistan; ^3^Department of Internal Medicine, Pikeville Medical Center, Pikeville, KY 41501, USA; ^4^Department of Internal Medicine, Maimonides Medical Center, Brooklyn, NY 11219, USA; ^5^Department of Internal Medicine, NYMC/Saint Mary and Saint Clare Hospital, Passaic, NJ 07834, USA; ^6^Department of Internal Medicine, Mercy Hospital St Louis, St Louis, MO 63141, USA; ^7^Department of Internal Medicine, University of Oklahoma Health Science Center, Oklahoma City, OK 73104, USA; ^8^Department of Cardiology, King George's Medical University, 226003 Lucknow, India; ^9^Division of Cardiology, University of Louisville School of Medicine, Louisville, KY 40202, USA

**Keywords:** disparity, race, major adverse cardiovascular events, GLP-1 RAs

## Abstract

**Background:**

Glucagon-like peptide-1 receptor agonists (GLP-1 RAs) have been shown to reduce major adverse cardiovascular events (MACEs) in patients with type 2 diabetes mellitus (T2DM) and high cardiovascular risk. However, the efficacy of GLP-1 RAs on the outcomes of MACEs across different racial and sex groups among patients with and without T2DM remains underexplored. Thus, this study aimed to evaluate the association between GLP-1 RAs and MACEs in patients with and without T2DM based on race and sex.

**Methods:**

We conducted a systematic literature search on the PubMed and Scopus databases, as well as ClinicalTrials.gov, for relevant randomized controlled trials (RCTs) from inception to July 5, 2025. Trials were eligible for inclusion if the included adults (≥18 years) had been randomized to a GLP-1 RA versus placebo group, and MACEs were reported as an outcome. Trials combining GLP-1 RAs with other investigational glucose-lowering agents were excluded. Risk ratios (RRs) and 95% confidence intervals (CIs) were pooled using a random-effect model, and a *p*-value of <0.05 was considered statistically significant.

**Results:**

Nine RCTs involving 81,266 patients were included in the analysis. The mean age of patients was 65 years. Compared with the placebo, GLP-1 RAs significantly reduced the risk of MACEs in males (RR, 0.82; 95% CI: 0.77–0.86; *p *< 0.001) and females (RR, 0.81; 95% CI: 0.75–0.88; *p* < 0.001). Meanwhile, across racial groups, GLP-1 RAs significantly reduced the risk of MACEs in Caucasian patients (RR, 0.87; 95% CI: 0.79–0.96; *p* < 0.001) compared with placebo. However, no significant difference was observed for the risk of MACEs in Black patients (RR, 1.05; 95% CI: 0.72–1.53;* p *= 0.80) when comparing GLP-1 RAs with placebo.

**Conclusion:**

This meta-analysis demonstrates that GLP-1 RAs significantly reduce the risk of MACEs in both males and females, as well as across various racial groups in patients with or without T2DM. However, the lack of significant benefit in Black patients suggests potential racial disparities in the enrollment and efficacy of GLP-1 RAs for cardiovascular outcomes.

## 1. Introduction 

Glucagon-like peptide-1 receptor agonists (GLP-1 RAs) have shown promising results in managing obesity and type 2 diabetes mellitus (T2DM) [1, 2, 3]. Several cardiovascular outcomes trials (CVOTs) have demonstrated that GLP-1 RAs significantly reduced the risk of three-point major adverse cardiovascular events (MACEs)—including cardiovascular death, nonfatal myocardial infarction, and nonfatal stroke—among diabetic and non-diabetic patients [1, 2, 3]. However, these trials have primarily emphasized overall treatment effects, with limited evaluation of differences based on sex, race, ethnicity, or geographic location. In addition, existing evidence suggests notable racial, ethnic, and geographic variations in cardiovascular and renal outcomes associated with these agents [4]. Hence, we aimed to evaluate the impact of GLP-1 RAs on MACE outcomes based on race and sex.

## 2. Methods

This meta-analysis was conducted and reported following the PRISMA (preferred reporting items for systematic review and Meta-analysis) 2020 guidelines. A comprehensive systematic literature search was conducted in PubMed, Scopus and ClinicalTrial.gov utilizing predefined MESH terms, coupled with the Boolean operators “AND” and “OR”. The search strategy included are (“Glucagon-Like Peptide-1 Receptor Agonists”[MeSH] OR “glucagon like peptide 1 receptor agonist*”[tiab] OR “GLP-1 receptor agonist*”[tiab] OR “GLP1-RA”[tiab] OR exenatide[tiab] OR liraglutide[tiab] OR semaglutide[tiab] OR dulaglutide[tiab] OR albiglutide[tiab] OR lixisenatide[tiab]) AND (“major adverse cardiovascular event*”[tiab] OR MACE[tiab] OR “myocardial infarction”[tiab] AND (race[tiab] OR racial[tiab] OR ethnicity[tiab] OR “African American”[tiab] OR Black[tiab] OR White[tiab] OR Caucasian[tiab] OR Asian[tiab] OR Hispanic[tiab] OR Latino[tiab]) AND (sex[tiab] OR gender[tiab] OR female[tiab] OR male[tiab] OR women[tiab] OR men[tiab]). We queried databases from inception to 5th July 2025. No language or time restrictions were applied.

Eligible studies included phase III, double-blind, placebo-controlled randomized controlled trials enrolling adults (≥18 years) that reported MACE. Non-randomized, non-placebo, or pediatric studies and animal trials were excluded. Two reviewers (VJ and YM) independently screened studies, extracted data, and resolved discrepancies through consensus.

We performed a conventional meta-analysis for the outcomes and adopted the DerSimonian and Laird random-effect model for the study variations. Outcomes were reported as pooled risk ratios (RR) and their corresponding 95% confidence intervals (CI). Statistical significance was met if the 95% CI did not cross the numeric “1” and the 2-tailed *p* value was <0.05. Heterogeneity was evaluated with I^2^ statistics. The risk of bias of the included studies was evaluated using the Cochrane Risk of Bias 2 (RoB 2) tool. Sensitivity and subgroup analyses were not conducted due to the limited number of eligible trials and the concise scope of this Brief Report. All the analyses were conducted using STATA version 17.1 (StataCorp, College Station, Texas, USA).

## 3. Results

**Fig. 1.**
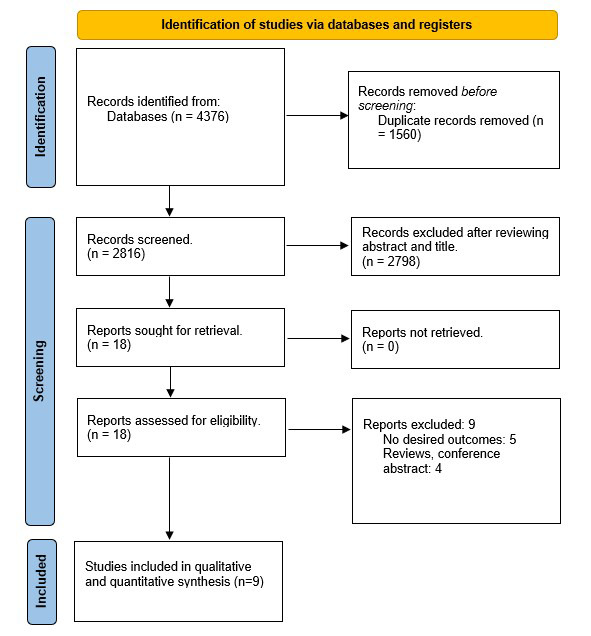
**PRISMA Flow chart of included studies**. PRISMA, preferred reporting items for systematic review and Meta-analysis.

The preliminary database search using the pre-specified keywords yielded 4376 articles, of which 1560 duplicate studies were excluded. Furthermore, 2798 studies were excluded based on title and abstract screening. Finally, 18 studies were sought for retrieval; of these, 9 were excluded because they lacked cardiovascular outcomes, were reviews, or were conference abstracts (Fig. 1). Risk of bias assessment indicated that all included studies had a low risk of bias (**Supplementary Fig. 1A,B**).

**Fig. 2.**
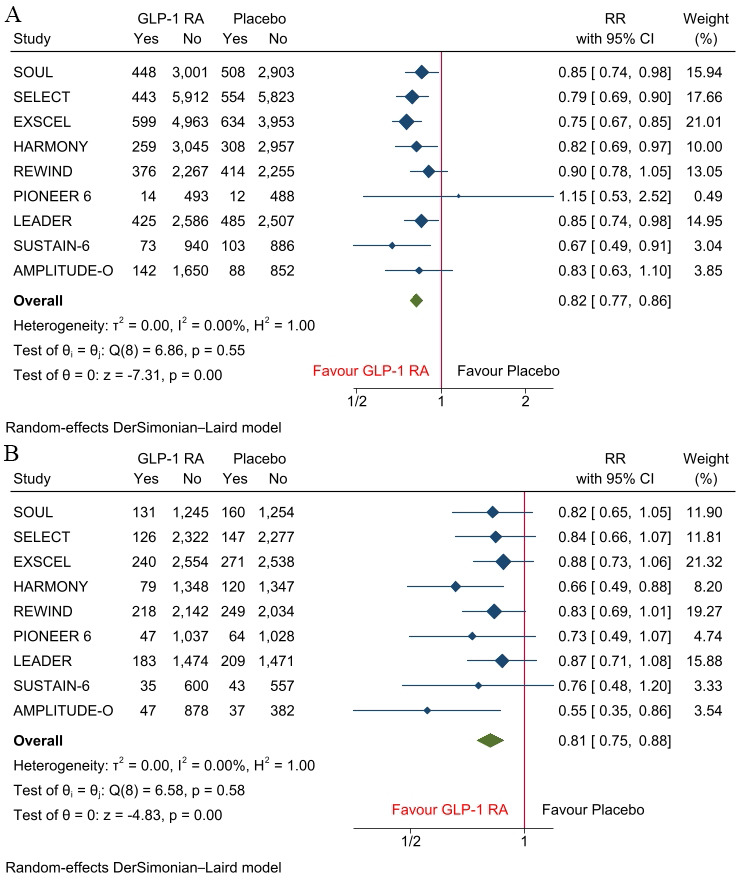
**Forest plots of MACE in (A) males and (B) females**. MACE, major adverse cardiovascular events; GLP-1 RA, Glucagon-like peptide-1 receptor agonist; RR, risk ratios.

**Fig. 3.**
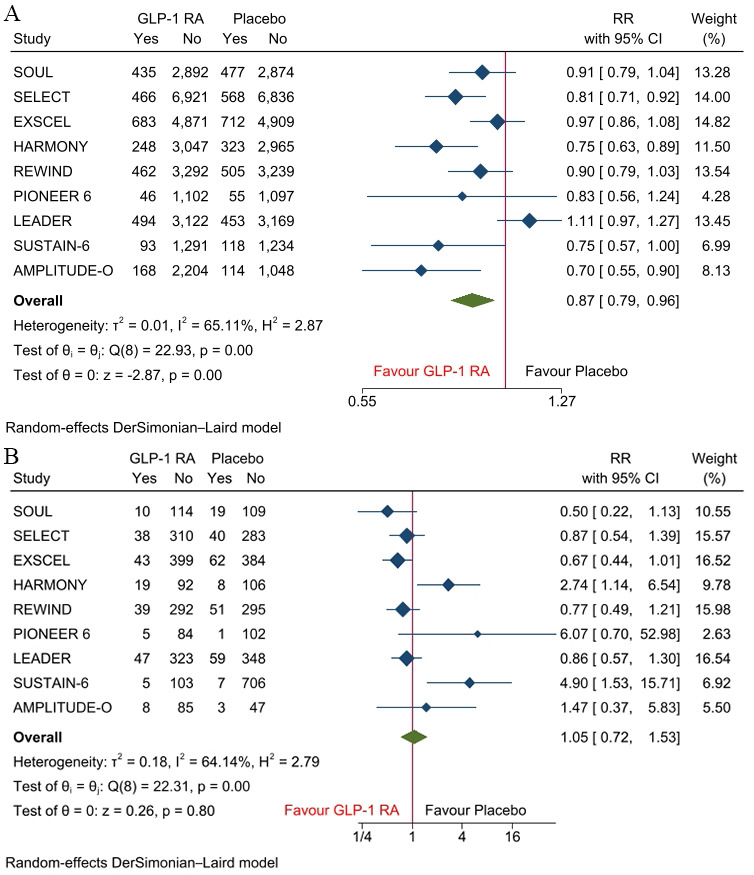
**Forest plots of MACE in (A) White patients and (B) Black patients**.

Nine RCTs involving 81,266 patients (41,288 receiving GLP-1 RAs and 39,978 receiving placebo) were included in the analysis [1, 2, 3, 5, 6, 7, 8, 9, 10] (Fig. 1). The mean age of patients was 65 years (Table 1). Compared with placebo, GLP-1 RAs significantly reduced the risk of MACE in males (RR, 0.82; 95% CI: 0.77–0.86; *p* < 0.001; I^2^ = 0%) and females (RR, 0.81; 95% CI: 0.75–0.88; *p* < 0.001; I^2^ = 0%) (Fig. 2A,B). These results indicate that both sexes derived a similar relative cardiovascular benefit from GLP-1 RA therapy. Across racial groups, GLP-1 RAs significantly reduced the risk of MACE in White patients (RR, 0.87; 95% CI: 0.79–0.96; *p* < 0.001; I^2^ = 65.11%) compared with placebo (Fig. 3A). However, no significant difference was observed in Black patients for the risk of MACE (RR, 1.05; 95% CI: 0.72–1.53; *p *= 0.80; I^2^ = 64.14%) when comparing GLP-1 RAs with placebo (Fig. 3B). This suggests potential racial differences in treatment response, with cardiovascular benefit observed in White patients but not in Black patients.

**Table 1.** **Baseline characteristics of studies included in the meta-analysis**.

Trials		Sample size	Age	Female	Diabetes duration	HTN	Follow up, years
SOUL	GLP1	4825	66.1 (7.6)	1376	14.7 (9–20.8)	4378	4.1
	Placebo	4825	66.1 (7.5)	1414	14.6 (8.9–20.8)	4381	
SELECT	GLP1	8803	61.6 (8.9)	2448	Non diabetic	NA	3.3
	Placebo	8801	61.6 (8.8)	2424	Non diabetic	NA	
EXSCEL	GLP1	7356	62.0 (2.0)	2794	12.0 (7.0–17.0)	NA	3.2
	Placebo	7396	62.0 (2.0)	2809	12.0 (7.0–18.0)	NA	
HARMONY	GLP1	4731	64.1 (8.7)	1427	14.1 (8.7)	4089	1.6
	Placebo	4732	64.2 (8.7)	1467	14.2 (8.9)	4095	
REWIND	GLP1	4949	66.2 (6.5)	2306	10.5 (7.3)	4605	5.4
	Placebo	4952	66.2 (6.5)	2283	10.6 (7.2)	4619	
PIONEER 6	GLP1	1591	66.0 (7.0)	507	14.7 (8.5)	NA	1.3
	Placebo	1592	66.0 (7.0)	500	15.1 (8.5)	NA	
LEADER	GLP1	4668	64.2 (7.2)	183	12.8 (8.0)	NA	3.8
	Placebo	4672	64.4 (7.2)	209	12.9 (8.1)	NA	
SUSTAIN-6	GLP1	1648	64.6 (7.4), 64.7 (7.1)	331, 304	14.3, 14.1	772 (93.5), 771 (93.8)	2.1
	Placebo	1649	64.8 (7.6), 64.4 (7.5)	342, 418	14.0, 13.2	756 (91.7), 760 (92.1)	
AMPLITUDE-O	GLP1	2717	64.6 (8.2)	925	15.6 (8.8)	2484	1.81
	Placebo	1359	64.4 (8.3)	419	15.1 (8.7)	1238	

GLP1, Glucagon-like peptide-1; HTN, hypertension.

## 4. Discussion

This meta-analysis confirms the cardioprotective effects of GLP-1 RAs, demonstrating a significant reduction in MACE. The inclusion of patients both with and without T2DM reinforces the growing evidence that GLP-1 RAs offer cardiovascular benefits beyond glucose control, potentially through mechanisms such as weight reduction, anti-inflammatory effects, and improved endothelial function [1, 2, 3, 5, 6, 7, 8, 9, 10, 11, 12]. Importantly, this study contributes to the existing literature by evaluating disparities in cardiovascular outcomes based on race and sex—an area that remains under-investigated, particularly among individuals with and without T2DM.

Sex-specific analyses showed that GLP-1 RAs conferred significant cardiovascular benefit in both males and females. Previous meta-analyses have reported similar findings, showing no significant interaction or sex differences in efficacy [11]. Although pharmacokinetic studies suggest minor sex-based differences in drug exposure and tolerability—particularly gastrointestinal side effects—these differences do not appear to influence cardiovascular outcomes [11]. These findings support the broad efficacy of GLP-1 RAs across sexes and reinforce their use in clinical practice without sex-based restriction.

However, racial subgroup analyses revealed a critical disparity. While White patients experienced a statistically significant reduction in MACE with GLP-1 RAs, no significant cardiovascular benefit was observed among Black patients. These findings raise the possibility of racial heterogeneity in treatment response or may reflect limitations in the available evidence base [12]. The lack of observed benefits in Black patients should be interpreted cautiously due to multiple contributing factors. Non-biological explanations are particularly compelling: Black participants have historically been underrepresented in large CVOTs of GLP-1 RAs, limiting statistical power to detect subgroup effects [1, 2, 3, 5, 6, 7, 8, 9, 10, 11, 12]. Additionally, residual confounding related to socioeconomic status, healthcare access, medication adherence, and comorbidity burden may have influenced outcomes [12]. Potential biological factors—such as genetic variability affecting GLP-1 receptor expression, drug metabolism, or pharmacodynamic response—could also play a role, though current evidence remains insufficient to confirm these mechanisms [12]. Therefore, without adequately powered and racially diverse trials, firm conclusions regarding the efficacy of GLP-1 RAs in Black patients cannot yet be drawn.

This meta-analysis has several limitations. First, it utilized trial-level rather than individual patient data, limiting the ability to adjust for confounders such as baseline risk, adherence, or social determinants of health. Second, subgroup analyses were constrained by the underrepresentation of certain populations, particularly Black patients, reducing the precision of these estimates. Inconsistencies in racial categorization across trials may also have introduced misclassification bias. Additionally, differences in cardiovascular outcome definitions, follow-up durations, and the specific GLP-1 RA agents used may have contributed to heterogeneity in the pooled results. However, we could not conduct sensitivity and additional subgroup analyses to address some of these concerns due to the limited data available in the trials and the brief scope of this report. These limitations highlight the need for more diverse, inclusive clinical trials and for future individual patient-level meta-analyses to better understand observed disparities.

## 5. Conclusion

While GLP-1 RAs reduce MACE risk across sexes and in White patients irrespective of diabetes status, the absence of observed benefit among Black patients highlights the need for future trials with more inclusive racial representation and stratified analyses. Targeted research is necessary to better understand potential biological, social, and systemic contributors to these disparities, ensuring that all patients can equitably benefit from advances in cardiovascular pharmacotherapy.

## Availability of Data and Materials

Data is provided within the article or **Supplementary Material**.

## Supplementary Material

Supplementary material associated with this article can be found, in the online version, at https://doi.org/10.31083/RCM45797.
