# Supplementary material for: Association Between Glucagon-Like Peptide-1 Receptor Agonists and Major Adverse Cardiovascular Outcomes Based on Race and Sex Among Patients With and Without Diabetes Mellitus: A Meta-Analysis of Nine Randomized Controlled Trials
Source: Rev Cardiovasc Med. 2026 Jan 21;27(1):45797. doi: 10.31083/RCM45797 (PMC12873659; doi:10.31083/RCM45797)
Supplement: Supplementary file 1 [file 2153-8174-27-1-45797-s1.zip › Supplementary Material.docx]

A) Risk of Bias Summary


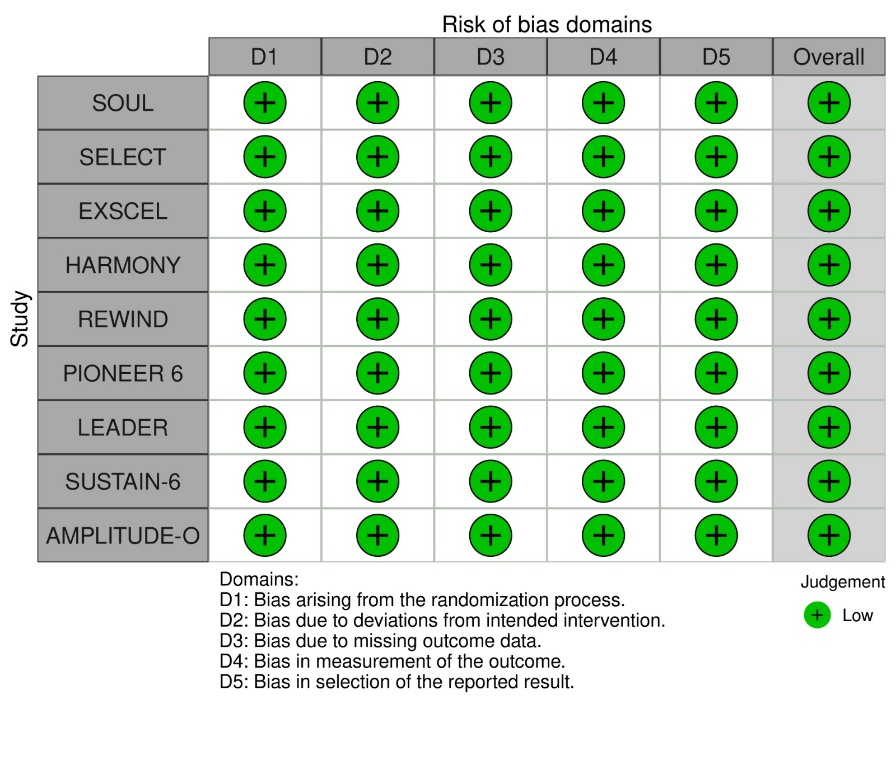


B) Risk of Bias Graph


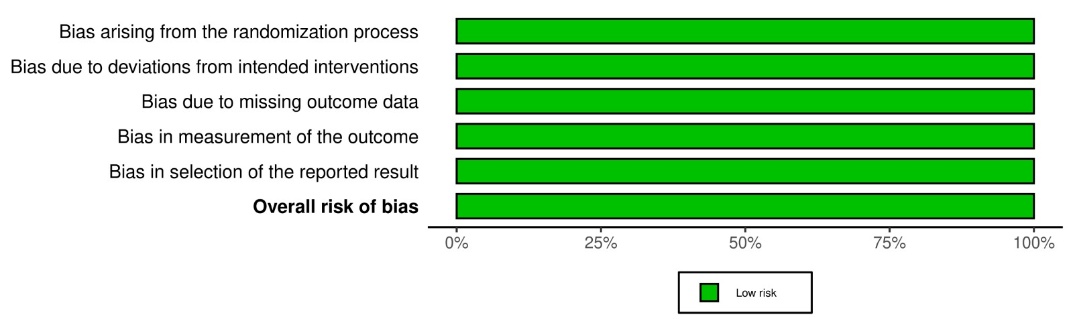


**Supplementary Fig. 1** Cochrane risk of bias including A) Risk of Bias summary, B) Risk of Bias graph
